# Efficacy and Safety of Transcutaneous Electrical Acupoint Stimulation for Improving Clinical Signs of Facial Skin Aging

**DOI:** 10.1111/jocd.70839

**Published:** 2026-04-05

**Authors:** Yi Luo, Hai Yu, Linxin Pan, Chun Yang, Jie Zhang, Tingting Yu, Wangjiao Li, Xiansheng Li, Jing Xu

**Affiliations:** ^1^ College of Acupuncture and Tuina Chengdu University of Traditional Chinese Medicine Chengdu China; ^2^ Department of Dermatology People's Liberation Army General Hospital of Western Theater Command Chengdu China; ^3^ Chengdu Tianfu Middle School Chengdu China

**Keywords:** aesthetic medicine, facial rejuvenation, noninvasive treatment, skin aging, transcutaneous electrical acupoint stimulation (TEAS)

## Abstract

**Background:**

Skin aging is a key focus in aesthetic and dermatologic medicine. Transcutaneous electrical acupoint stimulation (TEAS) is a noninvasive, acupuncture‐based technique that integrates traditional meridian theory with modern electrical stimulation. This study evaluated the clinical efficacy and safety of TEAS in improving the clinical signs of facial skin aging and promoting facial rejuvenation.

**Objective:**

This study aimed to evaluate the effects of TEAS on the clinical signs of facial skin aging.

**Methods:**

Ninety participants were randomly assigned to TEAS, acupuncture, or Sham‐TEAS groups. The TEAS group received 30‐min treatments, five times per week for four weeks. Primary and secondary outcomes were assessed using the VISIA analysis system and the FACE‐Q scale.

**Results:**

TEAS significantly reduced wrinkle scores at week 4 and week 8 compared with baseline (*p* < 0.001) and achieved greater improvement than acupuncture (*p* < 0.05). FACE‐Q scores increased significantly in the TEAS and acupuncture groups compared with Sham‐TEAS (*p* < 0.01), with greater improvement in Social Function in the TEAS group (*p* < 0.05). Both physician‐rated and self‐assessed GAIS and WSRS scores also improved in the TEAS group (*p* < 0.001).

**Conclusion:**

TEAS appears to be a safe, noninvasive intervention that is primarily effective in reducing wrinkle severity and improving patient‐reported outcomes, while showing limited effects on other VISIA parameters of facial skin aging.

**Abbreviations**
ECM: Extracellular Matrix
GAIS: Global Aesthetic Improvement Scale
TCM: Traditional Chinese medicine
TEAS: Transcutaneous electrical acupoint stimulation
TENS: Transcutaneous electrical nerve stimulation
VAS: Visual Analogue Scale
WSRS: Wrinkle Severity Rating Scale

## 1 Introduction

With socioeconomic development and evolving aesthetic preferences, demand for a youthful facial appearance has increased [1]. Facial anti‐aging has gradually replaced facial plastic surgery as the primary aesthetic pursuit. Facial anti‐aging includes preventive measures to delay aging as well as therapeutic methods aimed at improving and reversing aging, the latter of which is referred to as “rejuvenation” [2].

Aging is a natural physiological process, and the skin, as the largest organ of the human body and located at the outermost layer, serves as a barrier against pathogen invasion, water regulation, and electrolyte balance, and reflects the overall aging level of the body [3]. Skin aging primarily includes intrinsic aging (chronologic aging) and photoaging. Typical changes include laxity, wrinkles, dyspigmentation, reduced elasticity, and enlarged pores, which can negatively affect appearance and psychosocial well‐being. As people age, facial skin laxity and wrinkles become more pronounced, which significantly affect both appearance and self‐confidence.

Currently, skin anti‐aging strategies can be broadly categorized into two complementary approaches: (1) interventions targeting cellular and molecular drivers of skin aging, including cellular senescence and its associated signaling pathways; (2) therapeutic methods addressing age‐related structural changes by promoting tissue remodeling and restoring biomechanical support in the dermis and subcutaneous layers [4, 5]. In clinical practice, facial rejuvenation techniques are commonly categorized as surgical or non‐surgical. Non‐surgical approaches encompass topical and oral medications, injectable therapies, such as botulinum toxin and soft tissue fillers, energy‐based devices, including lasers and other device‐based rejuvenation technologies, and epidermal removal techniques like dermabrasion and chemical peels [6]. Surgical approaches encompass various lifting and wrinkle‐reduction techniques aimed at repositioning lax tissues and refining facial contours [7, 8]. Although these methods yield significant aesthetic outcomes, they are often associated with surgical discomfort, extended recovery periods, or potential adverse reactions [6, 8].

With the progress of medical technology and changes in public aesthetic preferences, people now tend to prefer safer, more natural, less invasive, and even noninvasive methods to achieve beauty. Compared with the commonly used traditional treatments, those based on traditional Chinese medicine (TCM) are relatively minimally invasive. Among these, acupuncture and electroacupuncture have been widely used in clinical practice for facial anti‐aging [9, 10]. However, traditional acupuncture still has certain limitations, such as patient anxiety, low acceptance rates, and the risk of bruising due to puncturing facial blood vessels. These issues highlight the need for a safer, more comfortable, and effective treatment method.

Transcutaneous electrical acupoint stimulation (TEAS) combines the principles of transcutaneous electrical nerve stimulation (TENS) with acupuncture points. It applies specific low‐frequency pulse currents to stimulate acupoints on the skin [11]. TENS has been shown to be effective in treating muscle atrophy and denervation by maintaining muscle mass, reducing atrophy, and promoting muscle reinnervation [12]. As a result, TENS has been widely used in populations unable to perform resistance training to prevent muscle atrophy and maintain muscle strength. Given its effectiveness in preventing muscle atrophy, it is worth investigating whether TEAS can also be effective in addressing facial aging issues, such as facial muscle relaxation and photoaging.

In this context, we conducted a randomized controlled trial to evaluate the efficacy of TEAS in improving the clinical signs of facial skin aging. We hypothesized that TEAS would lead to measurable improvements in wrinkle‐related outcomes compared with sham stimulation. This study aims to provide a new, noninvasive treatment option for facial skin aging and to offer scientific evidence for the application of TEAS in aesthetic medicine.

## 2 Methods

### 2.1 Study Design

This study was conducted in accordance with the Declaration of Helsinki and Good Clinical Practice guidelines. Approval was obtained from the Ethics Committee of a tertiary teaching hospital prior to participant recruitment. The study was conducted under the supervision of the ethics committee. All participants provided written informed consent before enrollment and were given sufficient time to consider their participation.

### 2.2 Participants

The inclusion criteria for this study were as follows: (a) Male or female subjects aged between 25 and 65 years old, who had varying degrees of skin laxity and wrinkles on their face, and were legally competent. (b) Subjects with Glogau photoaging grades II and III were included. (c) Participants were required to have the ability to understand and accept the related knowledge and risks associated with TEAS and facial acupuncture, and were required to sign the informed consent form. (d) Subjects were expected to be willing to cooperate with a long‐term satisfaction follow‐up survey.

The following exclusion criteria were applied: (a) participants who had undergone facial plastic and cosmetic treatment within 6 months; (b) subjects who received temporary skin filler treatment or energy‐based aesthetic treatment or surgical treatment on the face within 12 months before enrollment; (c) participants who had used OTC or prescription anti‐wrinkles to treat facial wrinkles within 28 days before enrollment or who planned to use these products during the course of the clinical study; (d) patients with serious underlying diseases; (e) patients with pacemakers, surgical implants, artificial hearts and lungs; (f) pregnant and lactating women and minors. Subjects who met any of the above criteria were excluded.

### 2.3 Randomization and Blinding

Eligible participants (*n* = 90) were randomly assigned (1:1:1) to one of three groups: TEAS, acupuncture, or Sham‐TEAS. Randomization was performed using a computer‐generated random number table created in Microsoft Excel by an independent researcher who was not involved in participant recruitment or assessment. Group assignments were concealed in sequentially numbered, opaque, sealed envelopes.

This study was participant‐ and assessor‐blinded. Participants and outcome assessors were blinded to group allocation. Treating physicians were not blinded because of the nature of the interventions; however, they were involved only in treatment delivery and had no role in outcome assessment or data analysis. To minimize information exchange and preserve blinding, participants received treatments in separate, enclosed rooms and at staggered time slots. Accordingly, the trial was participant‐ and assessor‐blinded, whereas treating clinicians were unblinded.

### 2.4 Intervention

#### 2.4.1 TEAS Group

**FIGURE 1:**
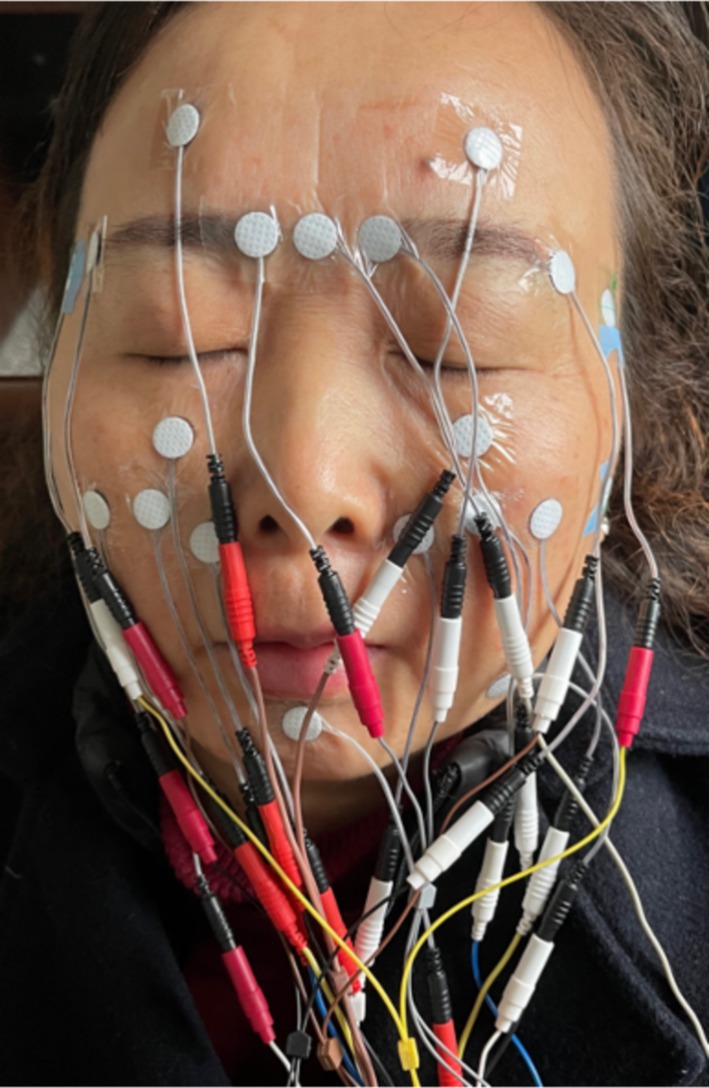
TEAS treatment.

Participants in the TEAS group received TEAS treatment (Figure 1) for 4 weeks (30 min per session, 5 sessions per week).

**FIGURE 2:**
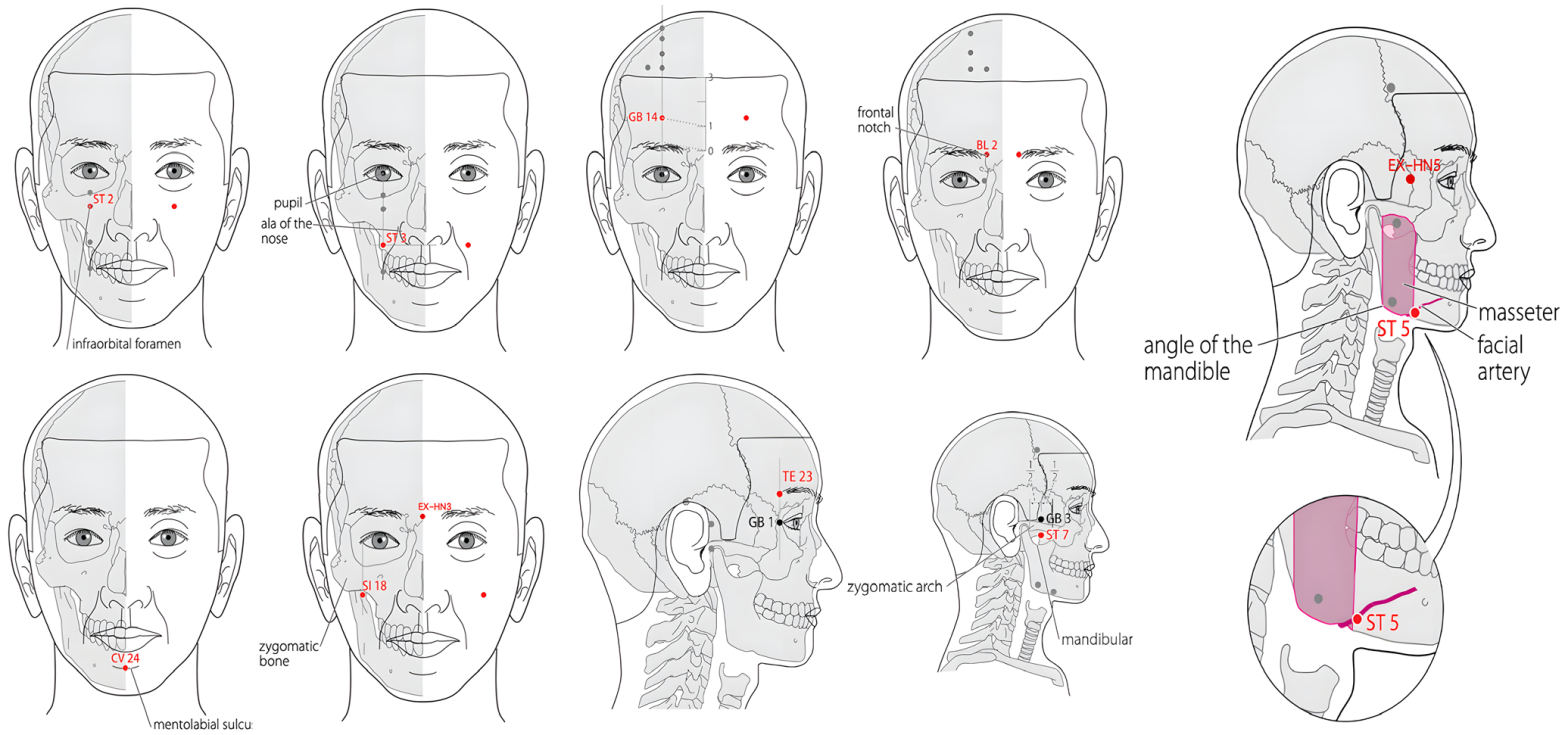
Scheme of selected acupoints.

- The Huatuo SDZ‐II electronic acupuncture device (Suzhou Medical Supplies Factory Co. Ltd.; Registration No.: Lu Ji Food and Drug Administration 20 201 201) was used.
- A 1 cm circular electrode patch (Shenzhen Chuangxinghe Technology Co. Ltd.; Product Registration No.: Yueshen Xibei 20 190 191) was applied to preselected acupoints.
- Acupoints included (Figure 2), CV24 (Chengjiang), LI20 (Yingxiang), ST2 (Sibai), ST3 (Juliao), SI18 (Quanliao), ST5 (Daying), ST7 (Xiaguan), BL2 (Cuanzhu), SJ23 (Sizhukong), EX‐HN5 (Taiyang), EX‐HN3 (Yintang), and GB14 (Yangbai).
- The device was operated in continuous wave mode. Stimulation frequency was gradually increased to 20 Hz during the initial minutes of each session according to participant tolerance, and intensity was adjusted to produce a perceptible but comfortable tingling sensation.

#### 2.4.2 Acupuncture Group

Participants in the acupuncture group received manual acupuncture at the same acupoints as the TEAS group, with the same treatment duration and frequency (30 min per session, 5 sessions per week, for 4 weeks).

- Sterile Qingling J15 acupuncture needles (Shizuoka Qingling Co. Ltd., Japan) were used.
- The procedure followed standard sterilization protocols, using 0.01% benzalkonium bromide solution and disposable sterile gauze.
- After facial cleansing, acupuncture was performed following TCM needling techniques.

#### 2.4.3 Sham‐TEAS Group

The Sham‐TEAS group underwent the same treatment protocol as the TEAS group, including identical electrode placement, session duration, and room setting. To enhance participant blinding, the device was switched on such that indicator lights and operational sounds were present, but no electrical stimulation output was delivered.

### 2.5 Outcomes Assessment

Before the study began, participants in the three groups completed baseline assessments of the primary and secondary outcome measures.

#### 2.5.1 Primary Outcomes

The VISIA skin analysis system is a tool that allows for the quantitative analysis of skin's pathological features. It has been extensively utilized in assessing indicators of facial rejuvenation. In this study, the sixth‐generation VISIA skin analysis system from Canfield Imaging Systems was used to measure facial rejuvenation metrics. The primary outcome was the change in VISIA scores for wrinkles, spots, texture, ultraviolet spots, pores, and red area at week 4 and week 8 compared with baseline.

#### 2.5.2 Secondary Outcomes

The FACE‐Q scale is primarily suitable for patients undergoing minimally invasive and open facial cosmetic surgery. This scale measures three main dimensions: facial appearance, health‐related quality of life, and adverse effects. Each dimension consists of multiple subscales. Our secondary outcomes of interest included changes in the three subscales of FACE‐Q: Age Visual Analogue Scale, Social Function, and Lines: Overall, as well as the Global Aesthetic Improvement Scale (GAIS) and the Wrinkle Severity Rating Scale (WSRS). The GAIS was assessed independently by two professional investigators (physicians) and by the participants themselves (self‐assessed). Furthermore, in the treatment group, we utilized a Visual Analogue Scale (VAS) to assess pain after each treatment session.

### 2.6 Safety and Adverse Events

Safety was assessed at each treatment session and follow‐up visit by documenting local dermatologic adverse events, including erythema, edema, pigmentation changes, bruising, and any other treatment‐related skin reactions. Adverse events were recorded by study personnel who were not involved in outcome assessment.

### 2.7 Statistical Analysis

SPSS version 27.0 was used for data analysis. Categorical variables were summarized as *n* (%). Continuous variables are presented as mean ± SD for normally distributed data and median (IQR) otherwise. Group comparisons at baseline were performed using one‐way ANOVA for continuous variables and χ^2^ tests for categorical variables, as appropriate. Repeated‐measures ANOVA was used to evaluate changes over time in VISIA parameters and FACE‐Q scores and to test time, group, and time × group effects. When the sphericity assumption was violated, the Greenhouse–Geisser correction was applied. When a significant interaction was detected, simple‐effects analyses were performed to examine between‐group differences at each time point and within‐group changes over time. WSRS and GAIS scores were compared among the three groups using the Kruskal–Wallis test. A two‐sided *p*‐value < 0.05 was considered statistically significant.

## 3 Results

### 3.1 Baseline Characteristics of the Participants

**FIGURE 3:**
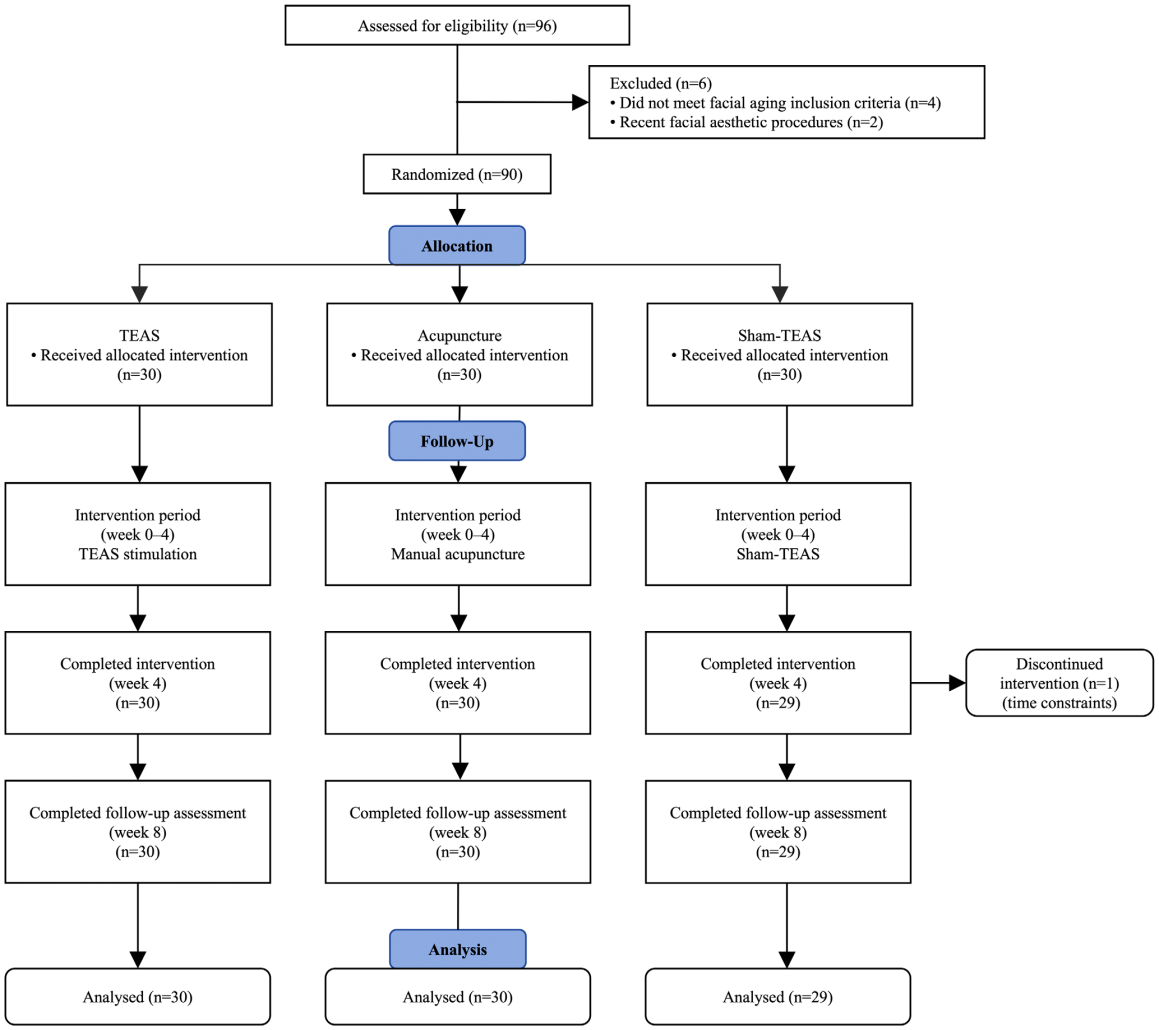
The study flowchart.

Of 90 participants randomized (30 per group, except 29 in Sham‐TEAS after one dropout due to time constraints) (Figure 3), baseline characteristics were comparable across the TEAS, Acupuncture, and Sham‐TEAS groups (Table 1). No significant differences were observed in age, gender distribution, VISIA skin scores (wrinkle, texture, red area, pores, and UV spots), or baseline FACE‐Q subscales among the three groups (*p* > 0.05 for all). No participant withdrew because of adverse effects during the study period, indicating good initial tolerability in all groups.

**TABLE 1:** Baseline characteristics of the participants.

Variable		TEAS (*n* = 30)	Acupuncture (*n* = 30)	Sham‐TEAS (*n* = 29)	statistic	*p*‐value
Age (years, ±)		42.87 ± 8.81	41.43 ± 8.52	42.21 ± 9.21	0.20	0.82
Gender (*n*, %)	Male	4(13.33%)	3(10.00%)	2(6.90%)	0.67	0.71
	Female	26(86.67%)	27(90.00%)	27(93.10%)		
VISIA data (±)	Wrinkle	21.98 ± 5.46	20.58 ± 4.76	23.46 ± 5.86	2.13	0.13
	Texture	1.32 ± 1.57	1.05 ± 0.88	1.04 ± 0.85	0.28	0.76
	Spots	25.32 ± 5.92	26.07 ± 7.7	23.24 ± 6.95	1.33	0.27
	Red area	26.73 ± 5.32	25.56 ± 4.98	25.43 ± 5.52	0.54	0.58
	Pores	12.21 ± 9.07	10.43 ± 6.16	10.44 ± 6.82	0.05	0.95
	UV spots	15.2 ± 9.02	12.25 ± 6.76	12.61 ± 6.69	0.78	0.46
FACE‐Q scale (±)	Age Visual Analogue Scale	1.43 ± 2.28	1.1 ± 2.19	1.07 ± 2.25	0.24	0.79
	Social Function	55.97 ± 6.53	54.17 ± 7.26	55.28 ± 7.28	0.5	0.5
	Lines: Overall	46.83 ± 10.08	44.67 ± 11.72	44.83 ± 12.04	0.34	0.71
WSRS (M, P25‐P75)		4(3,4.5)	3.5(3,4)	4(3,4.5)	1.78	0.41

### 3.2 Primary Outcomes

**FIGURE 4:**
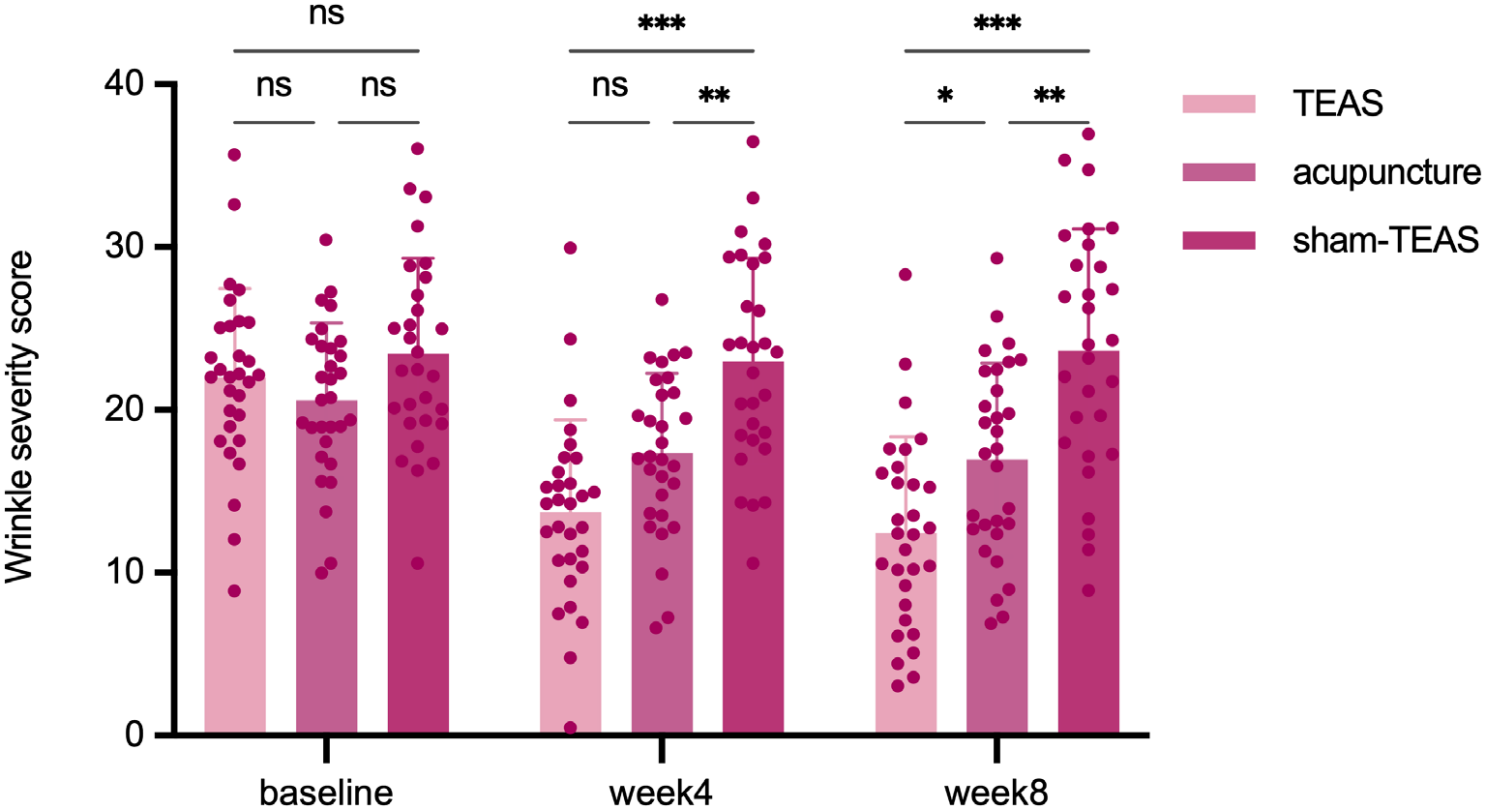
Comparison of wrinkle severity scores across groups. *P < 0.05, **P < 0.01, ns: No significance.

Repeated‐measures ANOVA (Tables 2 and 3) indicated that there was an interaction effect between time and group of wrinkle scores (*F* = 42.93, *p* < 0.001), as well as significant main effects of time (*F* = 110.98, *p* < 0.001) and group (*F* = 13.26, p < 0.001), suggesting that the intervention effects varied across time and groups. Further within‐group analysis showed that wrinkle scores decreased significantly over time in both the TEAS group (baseline: 21.98 ± 5.46; week 4: 13.71 ± 5.68; week 8: 12.45 ± 5.9, *p* < 0.001) and the Acupuncture group. However, no significant time effect was observed in the Sham‐TEAS group. Multiple comparisons at each time point (Table 4, Figure 4) indicated that at both week 4 and week 8, wrinkle scores in the TEAS group and the Acupuncture group were significantly lower than those in the Sham‐TEAS group (*p* < 0.001). Notably, the TEAS group exhibited a greater reduction than the Acupuncture group (week 4: I‐J = −3.63, [95% CI –7.20 to −0.06], *p* = 0.05; week 8 I‐J = −4.51, [95% CI –8.58 to −0.44], *p* = 0.03), suggesting superior wrinkle reduction. No interaction was observed in texture, spots, red area, pores, and UV spots (*p* > 0.05 for all), indicating that TEAS did not improve the above indicators.

**TABLE 2:** Changes in VISIA parameters over time.

VISIA parameter	Group	Baseline (Mean ± SD)	Week 4 (Mean ± SD)	Week 8 (Mean ± SD)	*F*	*p*‐value
Wrinkle	TEAS	21.98 ± 5.46	13.71 ± 5.68	12.45 ± 5.90	274.92	< 0.001***
	Acupuncture	20.58 ± 4.76	17.34 ± 4.89	16.96 ± 5.94	42.11	< 0.001***
	Sham‐TEAS	23.46 ± 5.86	22.97 ± 6.34	23.65 ± 7.46	1.60	0.21
Texture	TEAS	1.32 ± 1.57	1.34 ± 1.04	1.14 ± 0.98	1.33	0.27
	Acupuncture	1.05 ± 0.88	1.16 ± 0.97	1.20 ± 1.11	0.12	0.89
	Sham‐TEAS	1.04 ± 0.85	1.16 ± 1.16	1.04 ± 1.12	1.60	0.21
Spots	TEAS	25.32 ± 5.92	26.07 ± 5.05	24.42 ± 6.15	1.16	0.32
	Acupuncture	26.07 ± 7.70	25.22 ± 7.00	25.88 ± 7.10	0.42	0.66
	Sham‐TEAS	23.24 ± 6.95	22.29 ± 6.88	22.92 ± 7.39	0.48	0.62
Red area	TEAS	26.73 ± 5.32	25.63 ± 5.14	25.64 ± 4.87	1.32	0.27
	Acupuncture	25.56 ± 4.98	24.63 ± 4.67	24.51 ± 5.29	1.06	0.35
	Sham‐TEAS	25.43 ± 5.52	24.85 ± 5.91	24.26 ± 5.34	0.88	0.42
Pores	TEAS	12.21 ± 9.07	12.33 ± 9.89	11.86 ± 9.55	0.06	0.95
	Acupuncture	10.43 ± 6.16	10.14 ± 7.22	10.38 ± 8.12	0.30	0.75
	Sham‐TEAS	10.44 ± 6.82	9.99 ± 8.63	9.58 ± 7.87	1.28	0.28
UV spots	TEAS	15.20 ± 9.02	13.94 ± 7.97	12.15 ± 7.36	1.70	0.19
	Acupuncture	12.25 ± 6.76	11.21 ± 7.20	11.42 ± 7.91	0.84	0.44
	Sham‐TEAS	12.61 ± 6.69	10.36 ± 5.54	10.62 ± 5.98	1.45	0.24

*Note:* Significant differences are represented by asterisks: *p* < 0.05 (*), *p* < 0.01 (**), *p* < 0.001 (***).

**TABLE 3:** Repeated‐measures ANOVA results for VISIA parameters.

VISIA parameter	Time effect (*F*, *p*)	Group effect (*F*, *p*)	Time×Group interaction (*F*, *p*)
Wrinkle	*F* = 110.98, *p* < 0.001***	*F* = 13.26, *p* < 0.001***	*F* = 42.93, *p* < 0.001***
Texture	*F* = 0.42, *p* = 0.80	*F* = 0.77, *p* = 0.46	*F* = 1.34, *p* = 0.26
Spots	*F* = 0.76, *p* = 0.55	*F* = 2.16, *p* = 0.12	*F* = 0.30, *p* = 0.74
Red area	*F* = 0.09, *p* = 0.99	*F* = 0.60, *p* = 0.55	*F* = 3.02, *p* = 0.05
Pores	*F* = 0.27, *p* = 0.90	*F* = 0.44, *p* = 0.64	*F* = 1.23, *p* = 0.30
UV spots	*F* = 0.42, *p* = 0.77	*F* = 1.18, *p* = 0.31	*F* = 3.72, *p* = 0.03

**TABLE 4:** Multiple comparisons of wrinkle scores.

Time	Group(I)	Group(J)	I‐J	95% CI	*p*‐value
Week 4	TEAS	Acupuncture	−3.63	−7.20,–0.06	0.045*
		Sham‐TEAS	−9.26	−12.86,–5.66	< 0.001***
	Acupuncture	Sham‐TEAS	−5.63	−9.23,–2.03	< 0.001***
Week 8	TEAS	Acupuncture	−4.51	−8.58,–0.44	0.025*
		Sham‐TEAS	−11.20	−15.30,–7.09	< 0.001***
	Acupuncture	Sham‐TEAS	−6.69	−10.79,–2.58	< 0.001***

### 3.3 Secondary Outcomes

**FIGURE 5:**
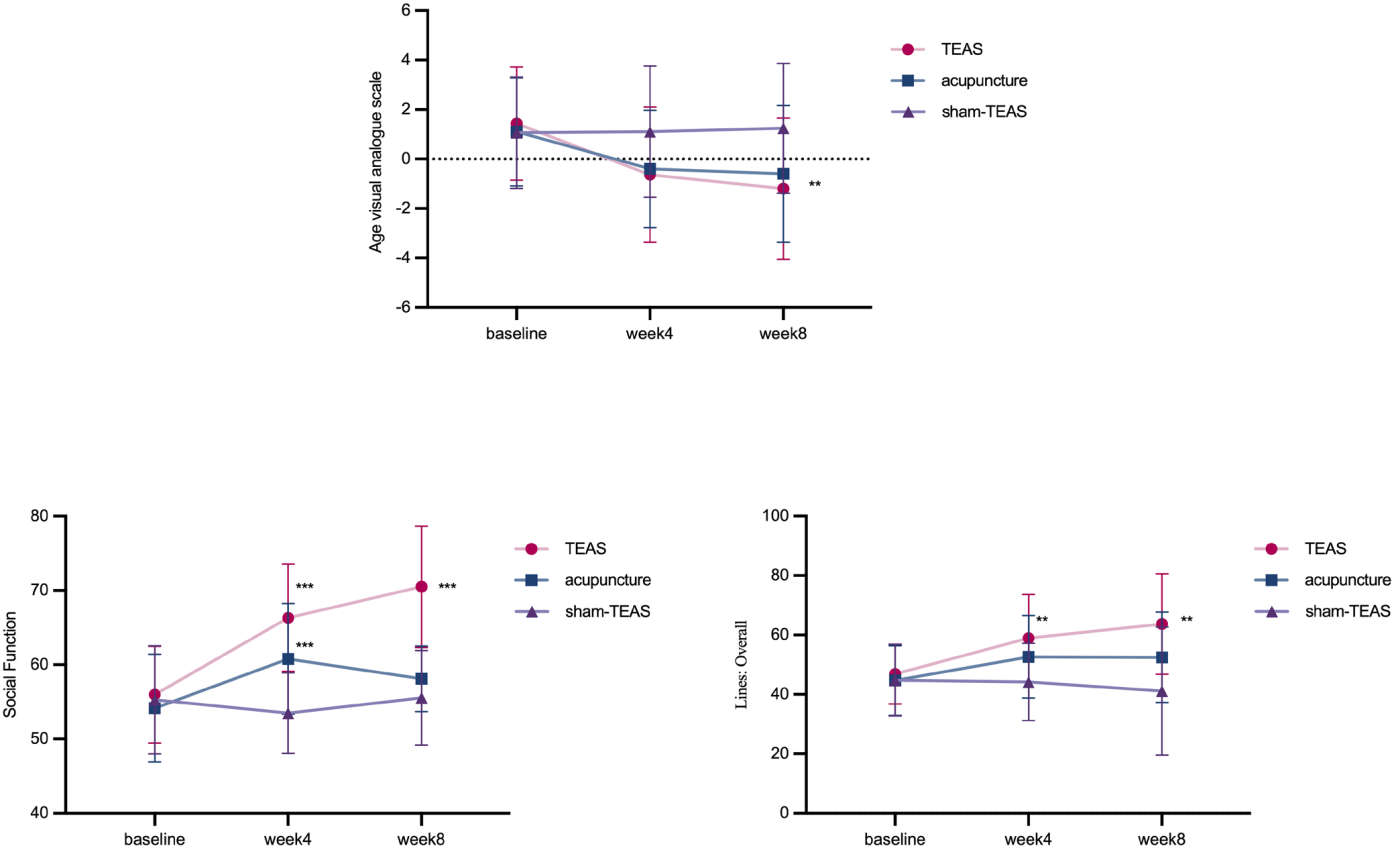
Changes in the FACE‐Q scale over time.

Tables 5, 6 and Figure 5 present a summary of the changes in self‐rating using the 3 subscales of the FACE‐Q scale before and after treatment in the three participant groups. Repeated measures ANOVA revealed significant interaction effects between time and group in all three subscales (Age Visual Analogue Scale: *F* = 20.47, *p* < 0.001; Social Function: *F* = 19.78, *p* < 0.001; Lines: Overall: *F* = 4.87, *p* < 0.001), indicating a differential change pattern among the groups. Both TEAS and Acupuncture groups showed significant improvements from baseline to week 8 (*p* < 0.01), whereas the Sham‐TEAS group showed no significant change (*p* > 0.05). The TEAS group showed significantly greater improvement in Social Function compared to the Acupuncture group (*p* < 0.05). No significant difference was found between TEAS and Acupuncture in the Age VAS or Lines subscales (*p* > 0.05).

**TABLE 5:** Changes in FACE‐Q scales over time.

FACE‐Q scale	Group	Baseline (Mean ± SD)	Week 4 (Mean ± SD)	Week 8 (Mean ± SD)	*F*	*p*‐value
Age Visual Analogue Scale	TEAS	1.43 ± 2.28	−0.63 ± 2.74	−1.20 ± 2.86	47.49	< 0.01**
	Acupuncture	1.10 ± 2.19	−0.40 ± 2.37	−0.60 ± 2.76	21.34	< 0.01**
	Sham‐TEAS	1.07 ± 2.25	1.10 ± 2.65	1.24 ± 2.63	0.37	0.7
Social Function	TEAS	55.97 ± 6.53	66.33 ± 7.21	70.50 ± 8.19	53.46	< 0.01**
	Acupuncture	54.17 ± 7.26	60.80 ± 7.45	58.10 ± 4.40	12.8	< 0.01**
	Sham‐TEAS	55.28 ± 7.28	53.48 ± 5.44	55.55 ± 6.36	1.66	0.20
Lines: Overall	TEAS	46.83 ± 10.08	58.93 ± 14.72	63.73 ± 16.91	12.75	< 0.01**
	Acupuncture	44.67 ± 11.72	52.63 ± 13.87	52.47 ± 15.21	3.95	0.02
	Sham‐TEAS	44.83 ± 12.04	44.21 ± 12.99	41.14 ± 21.60	0.56	0.57

**TABLE 6:** Repeated‐measures ANOVA results for FACE‐Q scales.

FACE‐Q scale	Time effect (*F*, *p*)	Group effect (*F*, *p*)	Time × group interaction (*F*, *p*)
Age Visual Analogue Scale	*F* = 62.09, *p* < 0.001***	*F* = 2.37, *p* = 0.099	*F* = 20.47, *p* < 0.001***
Social Function	*F* = 37.07, *p* < 0.001***	*F* = 24.84, *p* < 0.001***	*F* = 19.78, *p* < 0.001***
Lines: Overall	*F* = 8.18, *p* < 0.01**	*F* = 12.04, *p* < 0.001***	*F* = 4.87, *p* < 0.001***

Table 7 shows that both TEAS (*Z* = −4.786, *p* < 0.001) and Acupuncture (*Z* = −4.099, *p* < 0.001) significantly reduced wrinkle severity on the WSRS from baseline to week 8. No significant change was observed in the Sham‐TEAS group (*Z* = 1.732, *p* = 0.083).

**TABLE 7:** Comparison of WSRS M (P25‐P75).

Group		Before	Week 8	Wilcoxon test	
				*Z*	*p*‐value
TEAS		4(3,4.5)	3(2,4)	−4.79	< 0.001***
Acupuncture		3.5(3,4)	3(2,3.625)	−4.10	< 0.001***
Sham‐TEAS		4(3,4.5)	4(3,4.75)	1.73	0.08
Kruskal–Wallis test	H	1.78	8.85		
	df	2	2		
	*p*‐value	0.41	< 0.05*		

Table 8 and Table 9 show GAIS results. TEAS 2.5 (2,3) had better physician‐rated GAIS scores than Acupuncture 3 (2.5,4) (*p* = 0.002), and both were better than Sham‐TEAS 4 (4,4) (*p* < 0.001). Self‐assessed GAIS showed TEAS 3 (2,3) and Acupuncture 3 (3,4) superior to Sham‐TEAS 4 (3,5) (*p* < 0.001), with no significant difference between TEAS and Acupuncture (*p* = 0.311).

**TABLE 8:** Comparison of Physician GAIS M (P25‐P75).

Group		GAIS Score	Kruskal–Wallis test		
			Comparison	*Z*	*p* (Bonferroni‐adj.)
TEAS		2.5(2,3)	vs Acupuncture	−3.35	< 0.01**
Acupuncture		3(2.5,4)	vs Sham‐TEAS	−3.77	< 0.001***
Sham‐TEAS		4(4,4)	vs TEAS	−7.09	< 0.001***
Kruskal–Wallis test	H	50.31			
	df	2			
	*p*‐value	< 0.001***			

**TABLE 9:** Comparison of Self‐assessed GAIS M (P25‐P75).

Group		GAIS Score	Kruskal–Wallis test		
			Comparison	*Z*	*p* (Bonferroni‐adj.)
TEAS		3(2,3)	vs Acupuncture	−1.63	0.31
Acupuncture		3(3,4)	vs Sham‐TEAS	−2.55	< 0.05*
Sham‐TEAS		4(3,5)	vs TEAS	−4.16	< 0.001***
Kruskal–Wallis test	H	17.60			
	df	2			
	*p*‐value	< 0.001***			

### 3.4 Safety Analysis

Table 10 shows VAS scores. No pain was reported in the Sham‐TEAS group, whereas all subjects in the TEAS and Acupuncture reported experiencing a mild tingling sensation from the electrical acupoint stimulation, which they were able to tolerate. Across treatment sessions, the mean VAS score was 1.5 ± 0.63 in the TEAS group and 2.03 ± 0.76 in the Acupuncture group, ranging from 1 to 3 points, and no clinical intervention was required.

**TABLE 10:** Comparison of VAS scores.

Group	VAS Score (Mean ± SD)	95% CI	Comparison with TEAS *p*‐value
TEAS	1.50 ± 0.63	1.26–1.74	—
Acupuncture	2.03 ± 0.76	1.75–2.23	0.002*
Sham‐TEAS	0.00 ± 0.00	0.00–0.00	Not applicable

Overall, the interventions were well‐tolerated. No clinically meaningful local dermatologic adverse events (including erythema, edema, or pigmentation changes) were observed in the TEAS or Sham‐TEAS groups. In the Acupuncture group, two participants developed mild, transient bruising at needle sites, which resolved spontaneously without treatment, and no persistent pigmentation changes were noted.

## 4 Discussion

This randomized controlled trial investigated the effectiveness and safety of TEAS in improving the clinical signs of facial skin aging and promoting facial rejuvenation. The analysis showed that both TEAS (*F* = 274.92, *p* < 0.001) and Acupuncture (*F* = 42.11, *p* < 0.001) were effective in reducing wrinkle severity, with TEAS demonstrating more pronounced effects than the Sham‐TEAS group.

In addition, participants in the TEAS group also reported improvements in FACE‐Q and WSRS subscales. The study indicates partial alignment between clinician and subject perspectives—both groups identified Sham‐TEAS as the least effective on the GAIS scores. However, although clinicians perceived TEAS as superior to Acupuncture (*p* = 0.002), subjects (*p* = 0.31) viewed them as similarly effective. This discrepancy may be attributed to differences in professional judgment versus subjective experience, as well as possible placebo effects or blinding influencing participants' perceptions. These insights emphasize the importance of incorporating both objective (clinician) and subjective (patient) outcomes in aesthetic treatment evaluations. No treatment‐related adverse effects were observed, which supports the safety profile of TEAS as a noninvasive intervention.

The findings complement prior research on TEAS applied in neurological rehabilitation and general wellness [13, 14]. Although few studies have directly addressed its role in facial rejuvenation [8], the current results suggest that electrical stimulation at specific acupoints may help mitigate signs of skin aging, particularly those related to muscle laxity and decreased dermal elasticity [15]. Compared with traditional acupuncture [16], TEAS does not involve needle insertion, which could be more acceptable to certain patient populations and reduce potential complications.

The underlying biological mechanisms responsible for the observed improvements remain to be fully elucidated [9, 17]. It is assumed that TEAS promotes local microcirculation, which may facilitate nutrient and oxygen delivery to the skin, thereby enhancing collagen remodeling [13, 15, 18]. Acupoint stimulation [16, 18] might also trigger the release of biologically active compounds—such as neurotransmitters and growth factors—that are associated with dermal repair and anti‐aging processes. Moreover, the neuromuscular activation induced by electrical impulses could contribute to the improvement in facial muscle tone [19, 20, 21, 22, 23]. The beneficial effects of TEAS on facial skin aging may be related not only to improvements in local microcirculation and neuromuscular activation, but may also involve collagen remodeling, regulation of oxidative stress, and pathways related to angiogenesis [4, 24]. One of the key pathological features of skin aging is reduced collagen synthesis, increased collagen degradation, and the disorder of extracellular matrix (ECM) structure in the dermal layer [25]. Previous in vitro studies have shown that pulsed electrical stimulation can promote the proliferation of human dermal fibroblasts and upregulate the expression of growth factors such as PDGFA, FGF2, and TGF‐β1 [26]. It can also enhance the expression of α‐SMA and type I collagen [26], suggesting that electrical stimulation may promote fibroblast activation and enhance collagen deposition, thereby facilitating dermal remodeling and maintaining ECM homeostasis. In addition, oxidative stress is an important molecular mechanism for skin aging, especially photoaging [24, 27]. Excessive ROS can upregulate the expression of MMPs by activating the MAPK/AP‐1 and NF‐κB pathways, while suppressing TGF‐β/Smad signaling, ultimately leading to increased collagen degradation and ECM damage [28, 29]. Although there is currently insufficient evidence to directly demonstrate that TEAS regulates the ROS levels in facial skin, electrical stimulation has shown potential in tissue repair research for improving the local regenerative microenvironment, regulating inflammation, and promoting tissue regeneration. Therefore, TEAS may exert its effects partly by reducing ROS‐related damage [30, 31]. Furthermore, electrical stimulation may also activate angiogenesis‐related pathways [32]. Previous studies have shown that electrical stimulation can promote VEGF release and induce pre‐angiogenic responses in endothelial cells. Electric field stimulation may also activate eNOS through the PI3K/Akt pathway and increase NO production, whereas eNOS is an important effector molecule in VEGF‐mediated angiogenesis [33, 34, 35]. Therefore, TEAS may enhance oxygen and nutrient supply to skin tissues by promoting VEGF/eNOS‐related signaling, improving local perfusion and microvascular remodeling, thereby indirectly supporting collagen remodeling and tissue repair. However, these mechanisms remain hypothesis‐generating because no biochemical or histological data were collected in the present study.

In addition to the potential biological mechanisms discussed above, the acupoint selection in this study was also guided by traditional Chinese medicine (TCM) theory. From the perspective of TCM, the selection of facial acupoints in this study was primarily based on meridian theory and the concept of qi and blood nourishment [13]^.^ Classical TCM theory [36] states that the Yangming meridians govern the face and are characterized by abundant qi and blood, playing an important role in nourishing facial tissues. Therefore, several acupoints from the Stomach meridian were selected as core points because this meridian traverses key facial regions and may help regulate local qi and blood circulation. In addition, TCM theory considers impaired qi and blood circulation to be an important factor contributing to skin aging. When qi and blood circulation is insufficient, facial tissues may receive inadequate nourishment, leading to skin laxity and wrinkle formation [9]. Therefore, stimulation of facial acupoints to promote qi and blood circulation has traditionally been considered beneficial for improving facial skin condition. Furthermore, acupoints from several other meridians associated with facial regions were incorporated to form a multi‐meridian regulatory approach. According to meridian theory, these meridians collectively participate in regulating facial qi and blood circulation and tissue nourishment. This multi‐meridian acupoint combination provides the theoretical basis for the intervention in this study and reflects the integrative medicine perspective underlying the treatment strategy.

TEAS may be a promising option for patients seeking a noninvasive approach to reduce facial wrinkles. Its ease of use, favorable safety profile, and relatively low cost make it feasible for broader clinical adoption. As shown in our data, the majority of participants expressed satisfaction with the treatment outcomes, indicating that TEAS has the potential to address both objective and subjective expectations in aesthetic medicine. That said, the technique does not appear to significantly affect other skin parameters, such as pigmentation, pore visibility, or sun‐induced spots, as measured by VISIA. Therefore, TEAS may serve best as a targeted solution for fine lines and dynamic wrinkles, rather than a comprehensive anti‐aging approach. Combining TEAS with other modalities—for example, laser‐based treatments or injectables—may yield better overall results and is worth future exploration [6]. From a technological development perspective, TEAS offers notable advantages owing to its noninvasive nature, high safety profile, and operational simplicity, as it does not require needle insertion. In the future, with the advancement of wearable medical technologies and intelligent control systems, TEAS could be engineered into a portable, home‐use facial care device, enabling individualized, low‐risk facial rejuvenation management outside clinical settings. Such innovation would not only broaden its clinical applications in aesthetic medicine but also open new avenues for at‐home anti‐aging care, integrating safety, convenience, and accessibility into daily skincare routines.

To the best of our knowledge, this study is among the first randomized controlled trials to evaluate the application of TEAS in the field of facial rejuvenation. Importantly, its aesthetic efficacy was evaluated using a multi‐dimensional assessment framework, incorporating objective digital imaging (VISIA), clinician‐rated scales (WSRS, GAIS), and validated patient‐reported outcomes (FACE‐Q). This methodological integration enhances the reliability and translational value of the findings, offering a more holistic understanding of TEAS's clinical benefits in aesthetic medicine.

Several limitations should be acknowledged. First, the intervention period was relatively short, and long‐term follow‐up was not performed; therefore, the durability of the observed effects remains uncertain. Second, the study sample was predominantly composed of women and mainly included Asian participants with mild‐to‐moderate aging features, which may limit the generalizability of the findings to male populations, other ethnicities, different age groups, or individuals with more advanced facial aging. Third, only one TEAS protocol was evaluated, and the influence of different stimulation parameters on clinical outcomes was not assessed. In addition, although multiple measures were implemented to preserve blinding (staggered appointments, separate enclosed rooms, and an activated sham device with indicator lights and operational sounds), sensory differences between groups may have occurred. Notably, the Sham‐TEAS group reported VAS scores of 0, whereas the active TEAS and Acupuncture groups reported mild sensations and non‐zero VAS scores, which could have compromised blinding integrity and introduced expectation bias. At study completion, participants were informally asked about their perceived allocation, and most indicated that they were unaware of their assignment. Future trials should incorporate standardized blinding assessments and, where feasible, adopt low‐intensity sham stimulation to further reduce the risk of unblinding.

Future research should address these limitations by incorporating longer follow‐up, enrolling more diverse populations with balanced sex distributions, and comparing different stimulation settings to identify optimal treatment protocols. Comparative trials against other noninvasive modalities and studies evaluating combination strategies may further clarify the therapeutic scope of TEAS in aesthetic medicine and refine clinical indications [37].

## 5 Conclusion

TEAS appears to be a safe, noninvasive intervention that primarily improves wrinkle‐related outcomes in facial skin aging. Compared with sham stimulation, TEAS resulted in significantly greater reductions in wrinkle severity and improvements in clinician‐rated and patient‐reported measures. Benefits for other VISIA parameters were limited. TEAS was well‐tolerated, with no clinically meaningful dermatologic adverse events in the TEAS or Sham‐TEAS groups; two mild, transient bruising events occurred in the Acupuncture group. Further trials with longer follow‐up and more diverse populations are warranted to confirm durability and refine optimal protocols. These findings suggest that TEAS may serve as a valuable tool in the noninvasive aesthetic armamentarium. Further research is warranted to confirm its long‐term efficacy, explore its biological mechanisms, and assess its potential in combination with other cosmetic treatments.

## Author Contributions

**Yi Luo:** investigation, writing‐original draft preparation, writing – review and editing, and visualization. **Hai Yu:** investigation and writing – original draft preparation. **Linxin Pan:** visualization and data curation. **Chun Yang:** conceptualization and investigation. **Jie Zhang:** validation. **Tingting Yu:** formal analysis. **Wangjiao Li:** investigation. **Xiansheng Li:** investigation. **Jing Xu:** conceptualization, writing – review and editing, project administration, and funding acquisition.

## Funding

This work was supported by the National Natural Science Foundation of China (Grant No. 81774440) and the Provincial Natural Science Foundation of Sichuan (Grant No. 2024NSFSC0693).

## Ethics Statement

The study was approved by the Ethics Committee of the Affiliated Hospital of Chengdu University of Traditional Chinese Medicine (Approval No. 2022KL‐109). Informed consent was obtained from all participants. The trial was carried out in accordance with the principles of the Declaration of Helsinki. Additionally, it was registered with the International Traditional Medicine Clinical Trial Registry ITMCTR2024000600.

## Consent

Written informed consent for publication of identifying images was obtained from the participant.

## Conflicts of Interest

The authors declare no conflicts of interest.

## Data Availability

The datasets used and/or analyzed during the current study are available from the corresponding author on reasonable request.
